# AI-CONECT: Designing Responsible-AI-based Conversational Chatbots for Dementia Intervention

**DOI:** 10.1093/geroni/igaf122.3537

**Published:** 2025-12-31

**Authors:** Junyuan Hong, Wenqing Zheng, Han Meng, Siqi Liang, Liu Chen, Hiroko Dodge, Jiayu Zhou, Zhangyang Wang

**Affiliations:** University of Texas at Austin, Austin, Texas, United States; University of Texas at Austin, Austin, Texas, United States; Michigan State University, East Lansing, Michigan, United States; Michigan State University, East Lansing, Michigan, United States; Massachusetts General Hospital, Harvard Medical School, Boston, Massachusetts, United States; Massachusetts General Hospital, Harvard Medical School, Boston, Massachusetts, United States; Michigan State University, East Lansing, Michigan, United States; University of Texas at Austin, Austin, Texas, United States

## Abstract

Social isolation is recognized as a major modifiable risk factor for dementia. The I-CONECT clinical trial demonstrated that semi-structured, cognitively stimulating conversations can be an effective strategy for combating social isolation and cognitive decline among older adults with mild cognitive impairment (MCI). However, scaling such interventions is limited by the availability of trained human interviewers. To address this challenge, we developed the AI-CONECT Chatbot, a Responsible-AI system designed to implement the I-CONECT conversational intervention protocol using large language models (LLMs). This approach enables broad, scalable deployment without the human resource limitations of the original intervention. Our approach combines a privacy-preserving learning framework to automatically generate and evaluate protocol-compliant instructional prompts for LLMs with a novel evaluation strategy using “virtual users”—AI-based replicas of participants with MCI and participants with normal cognition (NC) from the I-CONECT clinical trial. In simulated conversations between chatbots and the virtual users, we found that optimized prompts can improve the user’s word ratio significantly over ChatGPT (ChatGPT: mean=0.235, std=0.059; ours: mean=0.489, std=0.065, p = 0.0004 by Mann-Whitney U test). The high word ratio implies better users’ conversational engagement. These findings suggest that LLMs, when carefully prompted, can engage older adults in cognitively stimulating dialogues consistent with established clinical interventions. Our preliminary results demonstrate a pathway for integrating responsible AI into enhancing cognitive functions, offering a practical and scalable alternative to human-facilitated conversational interventions. In our future work, we aim to improve the friendliness and other human-centered attributes of the chatbot.

